# Robust treatment planning in scanned carbon-ion radiotherapy for pancreatic cancer: Clinical verification using in-room computed tomography images

**DOI:** 10.3389/fonc.2022.974728

**Published:** 2022-08-29

**Authors:** Yohsuke Kusano, Hiroyuki Katoh, Shinichi Minohara, Hajime Fujii, Yuya Miyasaka, Yoshiki Takayama, Koh Imura, Terufumi Kusunoki, Shin Miyakawa, Tadashi Kamada, Itsuko Serizawa, Yosuke Takakusagi, Nobutaka Mizoguchi, Keisuke Tsuchida, Daisaku Yoshida

**Affiliations:** ^1^ Section of Medical Physics and Engineering, Kanagawa Cancer Center, Yokohama, Japan; ^2^ Department of Radiation Oncology, Kanagawa Cancer Center, Yokohama, Japan; ^3^ Accelerator Engineering Corporation, Kanagawa Office, Chiba, Japan; ^4^ Department of Heavy Particle Medical Science, Yamagata University Graduate School of Medical Science, Yamagata, Japan

**Keywords:** carbon-ion radiotherapy, gastrointestinal gas, scanning beam, pancreatic cancer, robust treatment plan, in-room CT, replacement

## Abstract

**Purpose:**

Carbon-ion beam (C-beam) has a sharp dose distribution called the Bragg peak. Carbon-ion radiation therapy, such as stereotactic body radiotherapy in photon radiotherapy, can be completed in a short period by concentrating the radiation dose on the tumor while minimizing the dose to organs at-risk. However, the stopping position of C-beam is sensitive to density variations along the beam path and such variations can lower the tumor dose as well as cause the delivery of an unexpectedly high dose to the organs at risk. We evaluated the clinical efficacy of a robust planning technique considering gastrointestinal gas (G-gas) to deliver accurate radiation doses in carbon-ion radiotherapy for pancreatic cancer.

**Materials and methods:**

We focused on the computed tomography (CT) value replacement method. Replacement signifies the overwriting of CT values in the CT images. The most effective replacement method for robust treatment planning was determined by verifying the effects of the three replacement patterns. We selected 10 consecutive patients. Pattern 1 replaces the CT value of the G-gas contours with the value of the region without G-gas (P1). This condition indicates a no-gas state. Pattern 2 replaces each gastrointestinal contour using the mean CT value of each contour (P2). The effect of G-gas was included in the replacement value. Pattern 3 indicates no replacement (P3). We analyzed variations in the target coverage (TC) and homogeneity index (HI) from the initial plan using in-room CT images. We then performed correlation analysis on the variations in G-gas, TC, and HI to evaluate the robustness against G-gas.

**Results:**

Analysis of variations in TC and HI revealed a significant difference between P1 and P3 and between P2 and P3. Although no statistically significant difference was observed between P1 and P2, variations, including the median, tended to be fewer in P2. The correlation analyses for G-gas, TC, and HI showed that P2 was less likely to be affected by G-gas.

**Conclusion:**

For a treatment plan that is robust to G-gas, P2 mean replacement method should be used. This method does not necessitate any particular software or equipment, and is convenient to implement in clinical practice.

## Introduction

The mortality rate remains high for pancreatic cancer. The standard treatment for unresectable locally advanced pancreatic cancer includes chemotherapy and chemoradiotherapy (1). Good outcomes with carbon-ion radiotherapy combined with gemcitabine (GEM), in particular, have been reported. Despite this, the overall survival rate is only a median of 21.5 months, and additional improvements in treatment outcomes are desired (2–5). In this context, Kawashiro et al. (3) reported that distant metastasis can be reduced by increasing the radiation dose, and studies on increasing the radiation dose have already been conducted (6). However, the effectiveness of such radiation dose increase will be lost unless the radiation dose is precisely delivered to the tumor. 

**Figure 1:**
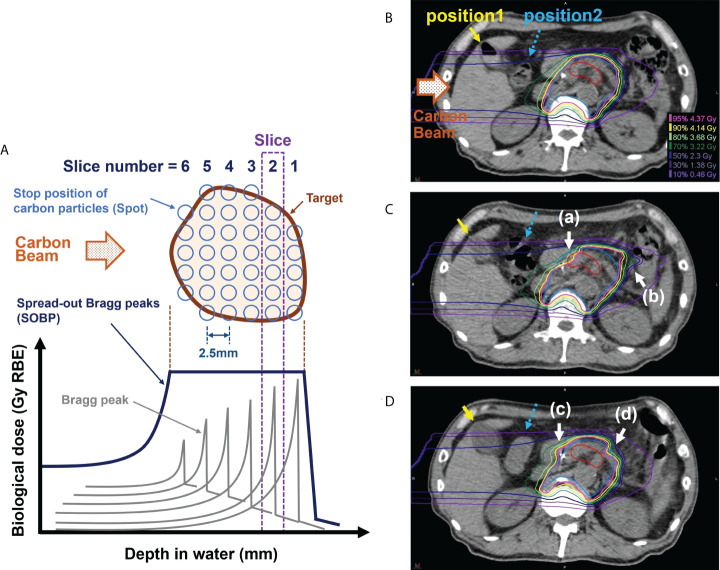
Effect of gastrointestinal gas (G-gas) on dose distribution in scanning carbon-ion radiotherapy for pancreatic cancer. **(A)** An illustration of the formation of the spread-out Bragg peaks (SOBP). **(B)** The dose distribution in the treatment planning computed tomography (CT) image in a sample case. **(C)** The dose distributions in the in-room CT images taken during the first irradiation. **(D)** The dose distributions in the in-room CT images taken during the ninth irradiation. The treatment of pancreatic cancer is conducted by performing irradiation in 12 fractions. During pancreatic cancer treatment, the stops of the carbon particles are determined at 2.5 mm water depth intervals. The red and blue contours indicate the gross tumor volume (GTV) and clinical target volume (CTV), respectively. The carbon beam direction is from the left side in each figure part.

A carbon-ion particle beam has a physical characteristic called the Bragg peak, which enables the delivery of a highly concentrated radiation dose to the tumor while reducing the radiation dose to adjacent organs (7, 8). In the treatment, the depth of water where the carbon-ion particles are stopped is determined in detail; moreover, an aggregate of Bragg peaks (spread-out Bragg peaks, SOBP) is formed and irradiated to the tumor (**Figure 1A**). During treatment planning, the water equivalent pass length to the stopping positions of carbon particles is calculated considering the presence of gastrointestinal gas (G-gas); moreover, the beam energy of carbon particles corresponding to the stopping positions and the number of particles corresponding to the doses are determined (positions 1 and 2 are shown in **Figure 1B**). The stopping position of carbon-ion particles is sensitive to G-gas variations along the beam path, and such variations can lower the tumor dose as well as cause the delivery of an unexpectedly high dose to the organs at risk (OAR). For example, when the beam path condition changes from no gas to gas, the carbon-ion particle stops at a deeper position than expected because of the shallower depth of the water than the planned stopping position. This decreases the tumor doses, thereby increasing the dose to OAR (**Figures 1B-D**). In a sample case of the first irradiation (**Figure 1C**), there was no G-gas in Position 1 and a significant increase in the amount of G-gas in Position 2. The impact of the computed tomography (CT) value change in Position 2 was significant, with CT values changing from −10 to −405 Hounsfield Unit (HU), thereby approaching the CT value of air. The density reduced, energy loss decreased, and carbon particles stopped at a deeper position than that during the treatment plan. Hence, the dose distribution of the proximal side was broken (**Figure 1C**-a), doses of gross tumor volume (GTV) and clinical target volume (CTV) were decreased, and dose to the distal side of the gastrointestinal tract was increased (**Figure 1C**-b). During the ninth irradiation (**Figure 1D**), the G-gas at Positions 1 and 2 was cleared. CT values changed from −415 HU to 38 HU in Position 1 and from −10 HU to 64 HU at Position 2. The density increased, energy loss increased, and carbon particles stopped at a position shallower than that during the treatment plan. Moreover, the doses to the duodenum increased (**Figure 1D**-c), and the dose of CTV decreased (**Figure 1D**-d). Hence, for minimizing the radiation dose to OARs and ensuring the adequate tumor dose, it is essential to prepare a treatment plan that is highly robust to the variations caused by G-gas in each radiation fraction.

G-gas, changes in the patient’s physique, and patient set-up error are some factors that can affect the tumor and OAR doses during treatment (9–16). Especially in pancreatic cancer, the target is surrounded by the gastrointestinal tract. Therefore, the dose distribution is more likely to be affected by the presence or absence of G-gas than when these organs are not involved. Kumagai et al. (10) evaluated the target coverage (TC) in carbon-ion radiotherapy of pancreatic cancer in relation to the positional changes of G-gas during irradiation using contrast-enhanced CT images and reported a reduction in TC. The ratio of the target volume irradiated to the irradiated volume greater than the evaluation dose is denoted by TC. Throughout the treatment period and irradiation, the position of the G-gas regions is unstable, and it is impossible to predict the G-gas position on each treatment day. Houweling et al. (13, 14) evaluated TC during the treatment period in pancreatic cancer using cone beam CT (CBCT) and found that TC decreased by 0.5% in X-ray radiotherapy of volumetric modulated arc therapy (VMAT), 8% in proton radiotherapy, and 10% in carbon-ion radiotherapy. The beam angle selection method (10–12) is an option for minimizing the effects of G-gas; however, the effects of G-gas cannot be avoided entirely. Additionally, in some cases, the beam angle affected by G-gas must be selected, such as in patients with kidney function impairment. Although the concept of online adaptive radiotherapy has been developed, several problems remain, such as excessive time consumption (17). Online adaptive radiotherapy is a technique in which the irradiation plan is modified according to the patient’s condition during each treatment procedure. In addition to the throughput, technical difficulties, such as the space allocation and magnetic field effects on equipment and beams and the implementation of in-room CT (irCT)-, CBCT-, or magnetic resonance imaging-based adaptive therapy for carbon-ion radiotherapy, will be major issues. In consideration of the hypoxic condition of the tumor, some studies have been conducted to reduce recurrence by controlling LET distribution in the tumor (18–21). In this case, as a nonuniform irradiation field was used for the treatment, high reproducibility of dose distribution during each treatment was required. Therefore, preparing a robust treatment plan that accounts for G-gas is critical.

We performed a preliminary analysis of the factors affecting the tumor dose during the treatment period in ten patients with pancreatic cancer; G-gas was one of the main factors (**Supplementary Figure 1**). If a robust treatment plan for G-gas can be prepared, tumor dose during the treatment period can be further improved. A robust planning method for G-gas has not been established in particle therapy. In this study, we focused on the effect of G-gas on dose distribution to help improve treatment outcomes in pancreatic cancer. Three G-gas replacement patterns were established and their effects were examined using irCT images taken during treatment. The most robust replacement method for G-gas was determined.

## Materials and methods

### Patient selection

**Figure 2:**
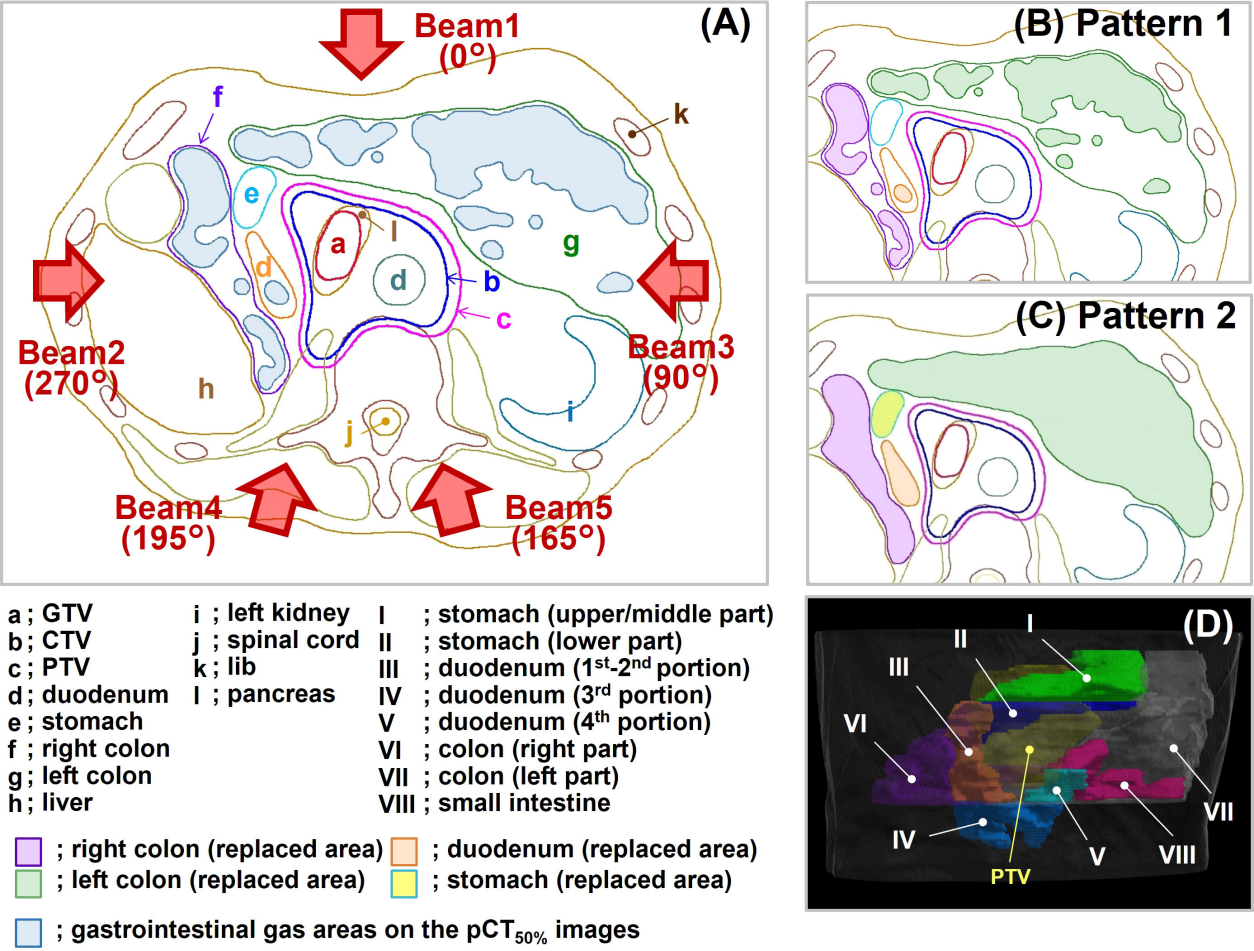
Replacement patterns and beam direction. **(A)** The supine position without replacement. This condition corresponds to Pattern 3. The blue-filled areas in the illustration represent gastrointestinal gas (G-gas). The gantry angle is indicated by a brown arrow. **(B)** The replacement condition in Pattern 1. **(C)** The replacement condition in Pattern 2. **(D)** Eight replacement regions in Pattern 2. GTV, gross tumor volume; CTV, clinical target volume; PTV, planning target volume; pCT_50%_ images, planning CT images of the maximum exhalation phase.

We selected ten consecutive patients who received carbon-ion radiotherapy for pancreatic cancer at our hospital from January 2019 to April 2020 (**Table 1**). This single-center study was conducted according to the guidelines of the Declaration of Helsinki, and approved by the Institutional Review Board of the Kanagawa Cancer Center (2019eki-106, August 30, 2021). Informed consent was obtained from all subjects, and their data were anonymized. Four-dimensional CT (4D-CT) images were obtained in the supine and prone positions for treatment planning because irradiation is performed using two fixed gantry ports from the horizontal and vertical directions (22). The irradiation angle (**Figure 2A**) was determined by combining the patient’s supine and prone positions as well as the treatment table’s rolling angle. The irCT images were obtained at least once every week during the treatment period considering each patient’s physical condition and X-ray exposure.

**Table 1:** Patient characteristics.

Patient	Age	Sex	Loc.	irCTscan times	Patient positioning	Volume (cm^3^)	
						GTV	CTV
1	60	M	H	6	SP	11.3	219.6
					PR	11.5	189.1
2	79	M	H	7	SP	10.7	238.8
					PR	11.1	231.4
3	54	F	H	6	SP	21.7	142.1
					PR	21.5	140.7
4	64	M	HB	5	SP	39.0	370.8
					PR	39.9	391.0
5	86	F	H	4	SP	10.7	112.7
					PR	11.1	102.0
6	78	F	H	4	SP	7.8	226.7
					PR	7.8	214.7
7	75	M	H	6	SP	26.3	184.1
					PR	26.2	213.3
8	65	F	HB	4	SP	10.9	288.4
					PR	11.3	279.1
9	72	M	H	7	SP	5.9	69.2
					PR	6.1	78.2
10	61	M	H	4	SP	25.0	274.0
					PR	28.8	291.8

Loc., location of tumor; irCT, in-room CT; GTV, gross tumor volume; CTV, clinical target volume; M, male; F, female; H, pancreatic head; HB, pancreatic head and body; SP, supine position; PR, prone position.

### Contouring of target and risk organs

The patients fasted at least 5 hours before the treatment planning CT (pCT) scan or treatment. An enema was performed if the patient had not defecated within the previous 24 hours. Patients were immobilized on the treatment table using patient immobilization devices (underneath: Blue BAG BodyFix, Elekta AB, Stockholm, Sweden, and upper surface: Shellfitter, Kuraray Co., Ltd., Tokyo, Japan). For all patients, 4D-CT scans were performed using a pCT scanner (Aquilion LB, Canon Medical Systems Corporation, Tochigi, Japan) under normal breathing. The raw data from 4D-CT scans are acquired at all respiratory timings based on the patient’s respiratory waveform. The CT images at ten respiratory timings (10% step) were created using 4D-CT raw data, with one respiratory cycle comprising 100% (0% and 100% were the maximum inhalation phase; 50% was the maximum exhalation phase), and those CT images are referred to as 4D-CT images. In this study, the CT images of the maximum exhalation phase were selected from the CT images of the ten phases and used for calculating the dose distributions and analyses. The CT images of the maximum exhalation phase in the pCT images are described as pCT_50%_ images.

GTV was delineated on the CT images at ten respiratory time points. The distance to GTV center of gravity was calculated at each respiratory time based on the maximum exhalation phase. Subsequently, the phase range in which the movement of GTV was within 5 mm was established. To create a uniform irradiation field, movement with respiratory gating “on” needs to be kept to within 5 mm (23). Due to inter-individual differences in maximum GTV and OAR movements, 4D-CT images are used to check each patient’s maximum GTV and OAR movements; subsequently, the phase range is determined. In our facility, the phase range is generally 30%–60%. Respiration speed is not constant between exhalation and inhalation; typically, the latter is faster than the former. Therefore, as tumor movement corresponds to respiratory movements, it is asymmetric in exhalation and inhalation. GTV movement can be controlled to within 3 mm in this situation.

CTV was defined as the GTV including a 5 mm margin and the locoregional elective nodal and neuro-plexus region (2, 3); the entire pancreas was included in the CTV as a preventive region regardless of the tumor site. The internal CTV (ICTV) was obtained by the summation of CTV within that phase range. The OARs (stomach, duodenum, colon, and small intestine) were delineated on the CT images in that phase range, and the summed OARs were defined as the planning organ-at-risk volume (PRV). Then, the planning target volume (PTV) was prepared by adding a margin of 3 mm to the ICTV, which was reduced when PTV was close to or overlapped the PRV.

### Replacement patterns of the gastrointestinal gas region

We focused on the CT value replacement method. The most effective replacement method was determined by verifying the effects of the three replacement patterns (**Figure 2**) using clinical data. Replacement implies that contours are drawn at the target locations of CT images and any CT values are assigned to each contour. This operation, which can be performed by the treatment planning system, can rewrite the CT values of the CT images. The carbon-ion scanning treatment planning system (Monaco for Carbon, Ver. 5.20, Elekta AB, Stockholm, Sweden) used in this study can achieve this same operation by replacing the value of the relative stopping power ratio (rSPR). The rSPR is calculated using the CT value and is equal to the CT value’s replacement (24–29). The rSPR of water and air are about 1.0 and 0.0, respectively. The rSPR corresponds to the relative electron density of photon radiotherapy. The following is a description of each pattern.

#### Pattern 1 (no-gas replacement condition)

In Pattern 1 (**Figure 2B**), the region of interest was set at a site without gas in each gastrointestinal tract, and the value of the rSPR was obtained on Monaco and used as the replacement value. The replacement region was defined by the gas contour (Gas_pCT50%_) delineated on the pCT_50%_ images. Gas_pCT50%_ was semiautomatically delineated using the threshold function of contouring software (MIM Maestro ver. 6.9.6, MIM Software Inc. Cleveland, OH, USA) with soft tissue conditions (window level = 40 HU, window width = 400 HU). The replacement values of Pattern 1 are shown in **Table 2**.

**Table 2:** Replacement values in the initial planning.

Patient	Patient positioning	Replacement value of rSPR in pCT_50%_ images, Pattern 1/Pattern 2			
		Small intestineAll/All	StomachAll/[U-M, L]	DuodenumAll/[1^st^–2^nd^, 3^rd^, 4^th^]	ColonAll/[Rt, Lt]
1	SP	1.03/1.03	1.04/[0.86, 0.84]	1.04/[1.02, 1.03, 1.03]	1.03/[0.86, 0.90]
	PR	1.03/1.03	1.03/[0.91, 1.03]	1.05/[1.02, 1.03, 1.03]	1.03/[0.89, 0.66]
2	SP	1.02/0.95	1.04/[1.04, 1.03]	1.05/[1.01, 1.02, 0.89]	1.03/[0.80, 0.74]
	PR	1.03/1.03	1.04/[0.99, 1.03]	1.04/[1.04, 1.03, 1.03]	1.03/[0.65, 0.98]
3	SP	1.04/1.04	1.05/[0.94, 0.94]	1.03/[1.02, 1.03, 1.02]	1.03/[0.95, 0.85]
	PR	1.04/1.03	1.03/[1.03, 1.03]	1.05/[1.02, 1.02, 1.02]	1.04/[0.98, 1.00]
4	SP	1.04/0.97	1.04/[1.03, 1.03]	1.03/[1.02, 1.02, 1.02]	1.03/[0.98, 0.89]
	PR	1.04/1.04	1.03/[1.02, 1.03]	1.04/[1.02, 1.02, 1.02]	1.03/[0.80, 0.90]
5	SP	1.04/1.03	1.04/[1.03, 0.90]	1.03/[0.97, 0.81, 1.01]	1.03/[0.76, 0.85]
	PR	1.04/1.04	1.03/[0.97, 0.97]	1.04/[1.04, 1.04, 1.04]	1.03/[0.90, 0.86]
6	SP	1.03/1.01	1.02/[1.01, 0.83]	1.03/[1.03, 1.00, 0.75]	1.02/[0.94, 1.01]
	PR	1.03/1.01	1.03/[0.99, 1.02]	1.04/[0.97, 0.97, 0.75]	1.02/[0.89, 0.73]
7	SP	1.05/1.04	1.04/[0.95, 0.97]	1.04/[1.03, 1.04, 1.04]	1.03/[0.65, 0.45]
	PR	1.03/1.03	1.04/[0.97, 1.03]	1.04/[0.97, 1.04, 1.04]	1.04/[0.49, 0.47]
8	SP	1.03/0.93	1.04/[0.71, 0.80]	1.03/[1.01, 1.03, 1.03]	1.02/[0.85, 0.96]
	PR	1.04/1.04	1.04/[0.73, 1.02]	1.03/[1.02, 1.02, 1.02]	1.03/[0.95, 0.78]
9	SP	1.04/1.03	1.04/[0.97, 0.99]	1.04/[1.03, 1.03, 1.04]	1.04/[0.94, 1.00]
	PR	1.03/1.03	1.03/[1.00, 1.01]	1.03/[1.00, 1.02, 1.02]	1.04/[0.93, 0.99]
10	SP	1.04/1.02	1.02/[1.02, 0.82]	1.05/[1.05, 1.05, 1.03]	1.02/[0.93, 0.91]
	PR	1.04/0.99	1.03/[1.00/1.00]	1.04/[0.96, 1.04, 1.03]	1.04/[0.92, 0.94]

rSPR, relative stopping power ratio; pCT_50%_ images, treatment planning CT images of the maximum exhalation phase; All, all regions of contour; U, gastric fundus; M, gastric body; L, pyloric zone; 1^st^–2^nd^, between the first and second (descending portion) portions of duodenum; 3^rd^, third portion (horizontal portion) of duodenum; 4^th^, fourth portion (ascending portion) of duodenum; Rt, right part; Lt, left part; SP, supine position; PR, prone position.

#### Pattern 2 (averaged-gas replacement condition)

In Pattern 2 (**Figure 2C**), the replacement regions were defined by gastrointestinal contours delineated on pCT_50%_ images. The stomach was divided into two regions comprising the upper/middle (**Figure 2D**-I) and lower parts (**Figure 2D**-II); the duodenum was divided into three regions comprising the 1^st^–2^nd^ (**Figure 2D**-III), 3^rd^ (**Figure 2D**-IV), and 4^th^ portions (**Figure 2D**-V); and the colon was divided into two regions comprising the right (**Figure 2D**-VI) and left parts (**Figure 2D**-VII). In addition to the small intestine (**Figure 2D**-VIII), eight replacement regions were set. Because G-gas accumulation in each organ is considered different for each patient and the degree of G-gas accumulation is considered to be different for each organ section, the replacement area was divided accordingly. The replacement value was defined as the mean rSPR of each replacement range. The regions were divided manually. The replacement values of Pattern 2 are shown in **Table 2**.

#### Pattern 3 (without replacement condition)

For Pattern 3 (**Figure 2A**), the optimization of dose distribution was performed without replacement.

### Initial plan

**Figure 3:**
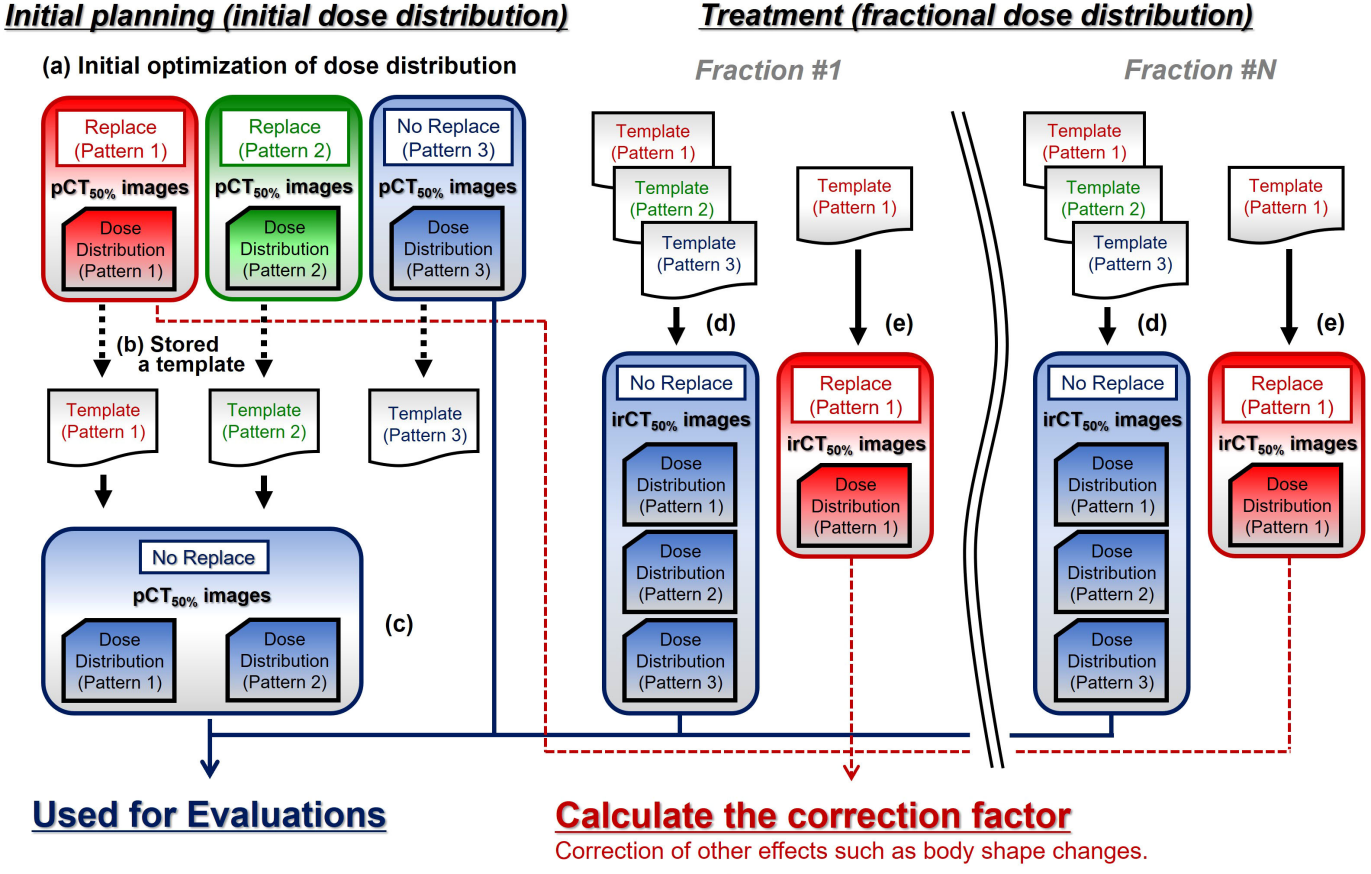
The calculation workflow of treatment planning computed tomography (CT) and fractional in-room CT (irCT) images. pCT_50%_ images, treatment planning CT images of the maximum exhalation phase; irCT_50%_ images, in-room CT images of the maximum exhalation phase; Replace, replace condition; No Replace, without replace condition; Template, irradiation conditions set (slice position, spot position in the plane of each slice position, and number of particles per spot etc.).

The replacement processing was performed using the abovementioned three patterns. With the gantry angle set to five directions as shown in **Figure 2A** (Gantry Angle: Beam 1 = 0°, Beam 2 = 270°, Beam 3 = 90°, Beam 4 = 195°, and Beam 5 = 165°), optimization of the initial dose distributions was performed with a single beam such that 95% of the prescribed dose covered PTV using pCT_50%_ images and contours (**Figure 3**-a). The prescribed dose per beam was set at a 4.6 Gy relative biological effectiveness-weighted absorbed dose (RBE), which is equivalent to a fraction dose for the pancreas (3, 30). The optimization-derived irradiation conditions were stored as templates (**Figure 3**-b). Subsequently, the five dose distributions of Patterns 1 and 2 were recalculated under no-replacement conditions (**Figure 3**-c) while maintaining the irradiation conditions determined in the optimizations. These results as well as the optimization results of Pattern 3 were used as initial planning results.

### Calculation of the dose distributions using fractional in-room CT images

In treatment, the patient’s irradiation position was set up by 2D–3D bone matching using front- and lateral-view X-ray images (2D) and pCT_50%_ images (3D) (31). The patient irradiation position was adjusted to the initial treatment plan based on the bone structure using the 2D and 3D images. The irradiation was performed using a high-speed scanning system for carbon-ion radiotherapy (CI-1000, TOSHIBA Corporation, Tokyo, Japan) (22, 32, 33). 4D-CT images were obtained using an irCT scanner while maintaining the patient set up at treatment. The irCT scanner is the same as the pCT scanner (22, 34). In this study, GTV, CTV, OARs, and Gas_irCT50%_ were delineated on the maximum exhalation phase in irCT images (irCT_50%_ images). The isocenter position was determined based on the markers projected onto irCT_50%_ images, and the fractional dose distributions were calculated for five gantry angles while maintaining the irradiation conditions determined in the initial plan using the templates (**Figure 3**-d).

### Correction of other effects

Changes in factors other than the effect of G-gas are included in the calculated dose distribution using irCT_50%_ images:


(1), $${\text{D}}_{\text{ir}}{\text{ = D}}_{\text{P}}{\text{ × f}}_{\Delta \text{Gas}}{\text{ × f}}_{\text{Other}}{\text{ (f}}_{\text{Other}}{\text{ = f}}_{\text{Str}}{\text{ × f}}_{\text{Pos}}{\text{ × f}}_{\text{Sur}}\text{ ×…})$$

where D_ir_ indicates the dose on irCT_50%_ images, and D_P_  indicates the dose on pCT_50%_ images, f_*Δ*Gas_ indicates changes in the effect of G-gas based on the initial plan; f_Other_ indicates factors other than G-gas; f_Str_ indicates the delineation error with morphological changes in the target; f_Pos_ indicates positional changes in the target; and f_Sur_ indicates the effects of changes in the patient’s physique. We calculated the dose distribution with Gas_irCT50%_ replaced by values without gas obtained on the external side of the gas region in addition to the calculations on pCT_50%_ images (Pattern 1 on **Figure 2B**). Using those dose distributions (**Figure 3**-e), we then calculated the correction factor based on the dose distribution on pCT_50%_ images for change in factors other than the effect of G-gas in each dose distribution on irCT_50%_ images:


(2) $${\text{k = D}}_{\text{ir(Rep)}}\;/{\text{ D}}_{\text{P(Rep)}}{\text{ =(D}}_{\text{P(Rep)}}{\text{ × f}}_{\text{Other}})\;/{\text{ D}}_{\text{P(Rep)}}{\text{= f}}_{\text{Other}}$$

(3), $${\text{D}}_{\text{ir(Cor)}}{\text{ = D}}_{\text{ir}}\;/{\text{ k =(D}}_{\text{P}}{\text{ × f}}_{\Delta \text{Gas}}{\text{ × f}}_{\text{Other}})\;/{\text{ f}}_{\text{Other }}{\text{= D}}_{\text{P}}{\text{ × f}}_{\Delta \text{Gas}}$$

where D_P(Rep)_ and D_ir(Rep)_ indicate the dose at the time of G-gas replacement on the beam pathway on the pCT_50%_ and irCT_50%_ images, respectively; k indicates the correction factor that converts the change in the factor of the dose other than G-gas in the irCT_50%_ images to the factor in the pCT_50%_ images; and D_ir(Cor)_ indicates the corrected dose on irCT_50%_ images. The values of k for each beam are shown in **Table 3**. The minimum and maximum values of k were 0.913 and 1.001 in the supine position and 0.830 and 1.010 in the prone position, respectively. The main determinant of k is believed to be changes in the tumor position (**Supplementary Figure 1**). These corrections enable the exclusion of changes in factors other than G-gas, thereby enabling the evaluation of the effect of G-gas alone. In this study, TC and homogeneity index (HI) were corrected by the correction factor of k.

**Table 3:** Changes in factors other than gastrointestinal gas (k).

Patient	Patient positioning	Value of k (min–max)				
		Beam 1 (0°)	Beam 2 (270°)	Beam 3 (90°)	Beam 4 (195°)	Beam 5 (165°)
1	SP	0.977–0.998	0.986–0.995	0.985–1.002	0.970–1.000	0.975–1.004
	PR	0.967–1.005	0.960–0.992	0.972–1.004	0.970–1.004	0.971–1.004
2	SP	0.961–0.984	0.966–1.003	0.963–1.004	0.953–1.002	0.953–1.010
	PR	0.988–1.008	0.980–0.999	0.950–0.988	0.990–1.006	0.993–1.010
3	SP	0.983–1.000	0.971–0.996	0.979–1.001	0.973–0.999	0.975–1.000
	PR	0.898–0.975	0.934–0.989	0.934–0.991	0.876–0.966	0.899–0.965
4	SP	0.978–1.011	0.965–0.985	0.975–0.991	0.987–1.002	0.995–1.002
	PR	0.980–0.991	0.958–0.992	0.965–0.989	0.960–0.963	0.984–0.993
5	SP	0.971–0.983	0.961–0.985	0.975–0.998	0.990–1.005	0.978–1.006
	PR	0.992	0.988	0.968	1.001	1.001
6	SP	0.944–0.977	0.948–0.980	0.951–0.983	0.945–0.981	0.948–0.980
	PR	0.926	0.911	0.919	0.902	0.901
7	SP	0.918–1.000	0.945–0.963	0.946–0.976	0.952–0.955	0.946–0.964
	PR	0.891–0.964	0.900–0.984	0.903–0.975	0.860–0.941	0.905–0.982
8	SP	0.992–0.999	0.991–0.996	0.985–0.994	0.959–0.997	0.953–0.993
	PR	1.008	1.007	1.007	0.994	0.993
9	SP	0.981–0.996	0.952–0.985	0.913–0.959	0.975–0.996	0.975–0.995
	PR	0.916–0.988	0.856–0.897	0.860–0.897	0.830–0.944	0.854–0.920
10	SP	0.927–0.993	0.972–1.002	0.969–1.005	0.980–1.008	0.982–1.004
	PR	0.990	0.973	0.982	0.982	0.990

min, minimum value; max, maximum value; SP, supine position; PR, prone position.

### Evaluation of CTV coverage and homogeneity variations from the initial plan

We analyzed and evaluated the variations in CTV coverage (TC_CTV_) and HI. For TC_CTV_, we evaluated the ratio of the volume irradiated by 95% or more of the prescribed dose (V_95%_). HI was then calculated using the following formula in accordance with ICRU83 (35):


(4), $${\text{HI =(D}}_{2\%}-{\text{D}}_{98\%})/{\text{D}}_{50\%}$$

where D_2%_ indicates the maximum dose, D_98%_ indicates the minimum dose, and D_50%_ indicates the median dose. For our analyses, we used the corrected values presented in the preceding paragraph. The variations in TC_CTV_ (ΔTC_CTV_) and HI (ΔHI) from the initial plan was defined using the following formulas:


(5) $$\Delta {\text{TC}}_{\text{CTV}}{\text{ = TC}}_{\text{CTV(irCT50\%)}}-{\text{TC}}_{\text{CTV(pCT50\%)}}$$

(6), $$\Delta {\text{HI = HI}}_{irCT50\%}-{\text{HI}}_{pCT50\%}$$

where TC_CTV(pCT50%)_ and HI_pCT50%_ , and TC_CTV(irCT50%)_ and HI_irCT50%_ are the values based on initial and fractional dose distributions, respectively.

### Evaluation between variations in G-gas volume and variations in CTV coverage as well as the HI during the treatment period

To examine the relationship between the volume variations in G-gas (ΔG-gas) during the treatment period, we analyzed the ΔTC_CTV_ and ΔHI against ΔG-gas. Even if the volume was the same, G-gas caused variations in CT values because of differences in moisture content. Accordingly, we believe that the rSPR was calculated using the CT value, thereby resulting in different contribution levels to the beam range. Therefore, gastrointestinal gas volume (Gas_R_) and ΔG-gas were defined using the formulas below, considering the effect on the beam range:


(7) $${\text{Gas}}_{\text{R }}=\;{\text{Gas}}_{\text{V}}\;/\text{ rSPR}$$

(8), $$\Delta {\text{G-gas = Gas}}_{\text{R(irCT) }}-{\text{ Gas}}_{\text{R(pCT)}}$$

where Gas_V_ indicates the volume of the gas contour delineated on the CT images (pCT_50%_ images: Gas_V_ = Gas_pCT50%_ on the beam pathway, irCT_50%_ images: Gas_V_ = Gas_irCT50%_ on the beam pathway). The mean rSPR of Gas_pCT50%_ and Gas_irCT50%_ were obtained using Monaco. The Gas_R _ of Beams 1, 2, and 3 for each patient are shown in **Table 4**. The Gas_R _ of Beams 4 and 5 were almost nil.

**Table 4:** Gastrointestinal gas volume considering the beam range in beam path (Gas_R_ ).

Patient	Patient positioning	Gas_R_ (cm^3^), pCT_50%_ images/irCT_50%_ images (min–max)		
		Beam 1 (0°)	Beam 2 (270°)	Beam 3 (90°)
1	SP	28.8/6.4–92.7	64.0/63.7–74.2	20.8/59.9–73.3
	PR	0.7/0.3–7.5	70.4/40.1–46.8	106.7/104.5–211.1
2	SP	90.0/37.6–139.1	49.1/38.2–89.3	151.6/55.7–180.9
	PR	3.3/0.0–42.2	86.8/12.5–71.1	25.5/33.8–174.9
3	SP	2.8/7.3–18.5	69.2/27.4–62.1	159.5/207.4–328.2
	PR	3.0/0.8–4.2	28.7/8.9–36.9	21.0/45.1–81.0
4	SP	3.3/14.1–49.3	1.3/22.8–42.9	23.3/16.0–75.5
	PR	0.3/0.2–0.4	4.5/1.0–6.0	30.2/32.4–71.3
5	SP	6.0/0.0–2.1	52.9/1.9–5.0	95.0/12.2–117.0
	PR	1.0/0.4	2.2/1.0	25.2/34.0
6	SP	54.9/12.4–44.6	34.9/4.7–28.0	32.0/43.8–207.4
	PR	26.2/32.0	28.8/6.6	102.5/43.3
7	SP	442.4/52.8–60.6	142.9/36.3–41.1	353.7/22.1–47.7
	PR	7.7/2.3–4.8	15.6/1.3–0.5	20.6/25.9–30.2
8	SP	21.4/10.6–400.1	65.6/32.6–46.0	69.8/42.3–50.5
	PR	0.4/0.2	25.1/20.1	195.8/11.5
9	SP	1.7/1.2–3.9	6.2/16.9–52.1	2.9/16.4–52.1
	PR	1.4/0.0–2.5	12.8/7.4–39.8	2.7/4.3–102.7
10	SP	84.0/5.9–70.3	24.4/7.8–50.4	73.0/41.6–70.5
	PR	9.7/0.0	40.9/72.1	43.3/31.1

Gas_R_, gastrointestinal gas volume considering the beam range in beam path; pCT_50%_ images, treatment planning CT images of the maximum exhalation phase; irCT_50%_ images; in-room CT images of the maximum exhalation phase; min, minimum value; max, maximum value; SP, supine position; PR, prone position. The Gas_R _ of Beams 4 and 5 were almost nil.

### Statistical analysis

The differences between the three replacement techniques were evaluated using CT images from the same patient in this study. There was no normality in each data set. The Friedman test was used because this is a three-group evaluation of quantitative data. Since the comparison of the three groups would be evaluated thrice, the obtained p values were multiplied by three using the Bonferroni method. Finally, we conducted significance tests with the p value set to <0.05. The statistical software used was SPSS (IBM SPSS Statistics, version 26.0, IBM, Inc., Armonk, NY, USA).

In the evaluation between variations in G-gas volume and *Δ*TC_CTV_ and *Δ*HI, the results were linearly fitted by the least-squares method; moreover, the correlation analysis (R^2^) was performed.

In the evaluation of the positional changes of gastrointestinal gas, we used a Wilcoxon signed-rank test to evaluate two groups without normality, and then we conducted significance tests with the p value set to <0.05.

## Results

### Evaluation of variations in CTV coverage and the HI from the initial plan

**Figure 4:**
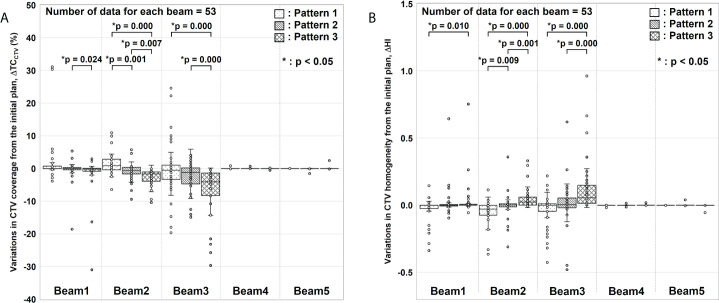
Evaluation results for variations in clinical target volume (CTV) coverage (ΔTC_CTV_) and homogeneity index (ΔHI). **(A)** ΔTC_CTV_ and **(B)** ΔHI values from the initial plan. For each gantry angle, the median, first quartile, third quartile, maximum and minimum values, and outliers for ΔTC_CTV_ and ΔHI are presented in a box-and-whisker plot. These results were corrected for changes in factors other than gastrointestinal gas. Statistical analyses were then performed using the Friedman test. The symbol * indicate that there is a significant difference between them.

**Figure 4** shows the box-and-whisker plots of ΔTC_CTV_ and ΔHI from the initial plan.

In ΔTC_CTV_ (**Figure 4A**), Beams 4 and 5 were almost nil because of the absence of interference from G-gas. For Beam 1, a significant difference was observed between Patterns 2 and 3. For Beam 2, variations were significantly fewer for Pattern 2 than for Patterns 1 and 3. For Beam 3, a significant difference was observed between Patterns 1 and 3 and between Patterns 2 and 3. In particular, a remarkable difference was observed for Beam 2 and 3 with which a major change was observed in the G-gas volume. These results demonstrate that the dose distribution is best optimized with G-gas replaced. Although no statistically significant difference was found between Patterns 1 and 2 except for Beam 2, ΔTC_CTV_, including the median value, tended to be fewer with Pattern 2.

The same tendency was observed for ΔHI (**Figure 4B**); ΔHI with Beams 4 and 5 were almost nil. With Beams 1 to 3, variations were significantly fewer for Pattern 2 than for Patterns 1 and 3.

### Evaluation of the variations in G-gas volume and the variations in CTV coverage and the HI during the treatment period

**Figure 5:**
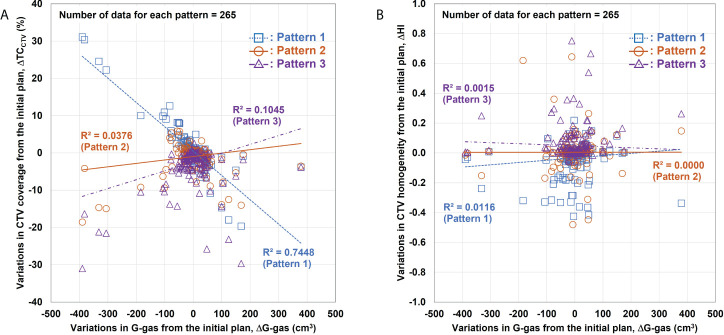
Relationship between variations in the gastrointestinal gas (ΔG-gas) volume and the variations in the clinical target volume (CTV) coverage (ΔTC_CTV_) and homogeneity index (ΔHI). **(A)** Relationship between ΔG-gas and ΔTC_CTV_ (V95%); **(B)** ΔHI. The horizontal axis shows the volume variation in G-gas from the initial plan. The effect of the beam range is considered in ΔG-gas. The vertical axes in **(A)** and **(B)** show the variations in TC_CTV_ and HI from the initial plan, respectively. The smaller the gradient, the smaller the effect of G-gas.

**Figure 5** shows the relationship between the volume variation in G-gas (ΔG-gas) from the initial plan as well as the variation in TC_CTV_ (ΔTC_CTV_) and HI (ΔHI) from the initial plan for each replacement pattern. Linear fitting was performed for each replacement pattern. In this figure, a steep slope indicates the large influence of G-gas. Actual dose distribution is significantly impacted by changes in G-gas, as indicated by a high R^2^ correlation coefficient. For TC_CTV_, Pattern 1 has a large absolute value of a linear fitting slope and a large R^2^, indicating that it is greatly affected by G-gas, whereas Pattern 2 has a slope that is closest to zero and a very small R^2^, indicating that it is less affected by G-gas (**Figure 5A**). For HI, the absolute value of the slope is small for all patterns; however, the value of R^2^ is the smallest for Pattern 2, indicating that the effect of G-gas is also small (**Figure 5B**).

## Discussion

In this study, we proposed a method to robustly plan for the effect of G-gas in the treatment of pancreatic cancer by carbon-ion scanning irradiation, and we evaluated the validity of the method based on clinical data. We found that the replacement area in Pattern 2 is optimal for setting the replacement region and that replacing the mean value with Pattern 2 was effective.

Using the proposed Pattern 2 mean value replacement method, the replacement value of G-gas was determined for each patient, and individual differences in the incidence of G-gas were considered. Furthermore, the replacement value was determined for each organ and section, even for the same organ, thereby considering differences in the degree of G-gas accumulation. Although the replacement regions were complicated, we were able to successfully use this method for routine treatment planning without compromising throughput. However, as shown in **Figure 4**, dose variations were observed in some cases even when the mean value replacement method was used. This may be because the location or amount of G-gas varied significantly from the treatment plan. Therefore, when implementing this method in clinical practice, it is necessary to carefully observe the location and amount of G-gas in the X-ray images obtained for 2D–3D bone matching at the time of each treatment before irradiation. If the variations in the location or proportion of G-gas are significant, the precautionary measures, such as routine verification of dose distributions using irCT images, seem to be necessary. Moreover, there is a need for an institutional protocol for the dividing method of the small intestine to minimize individual differences.

**Figure 6:**
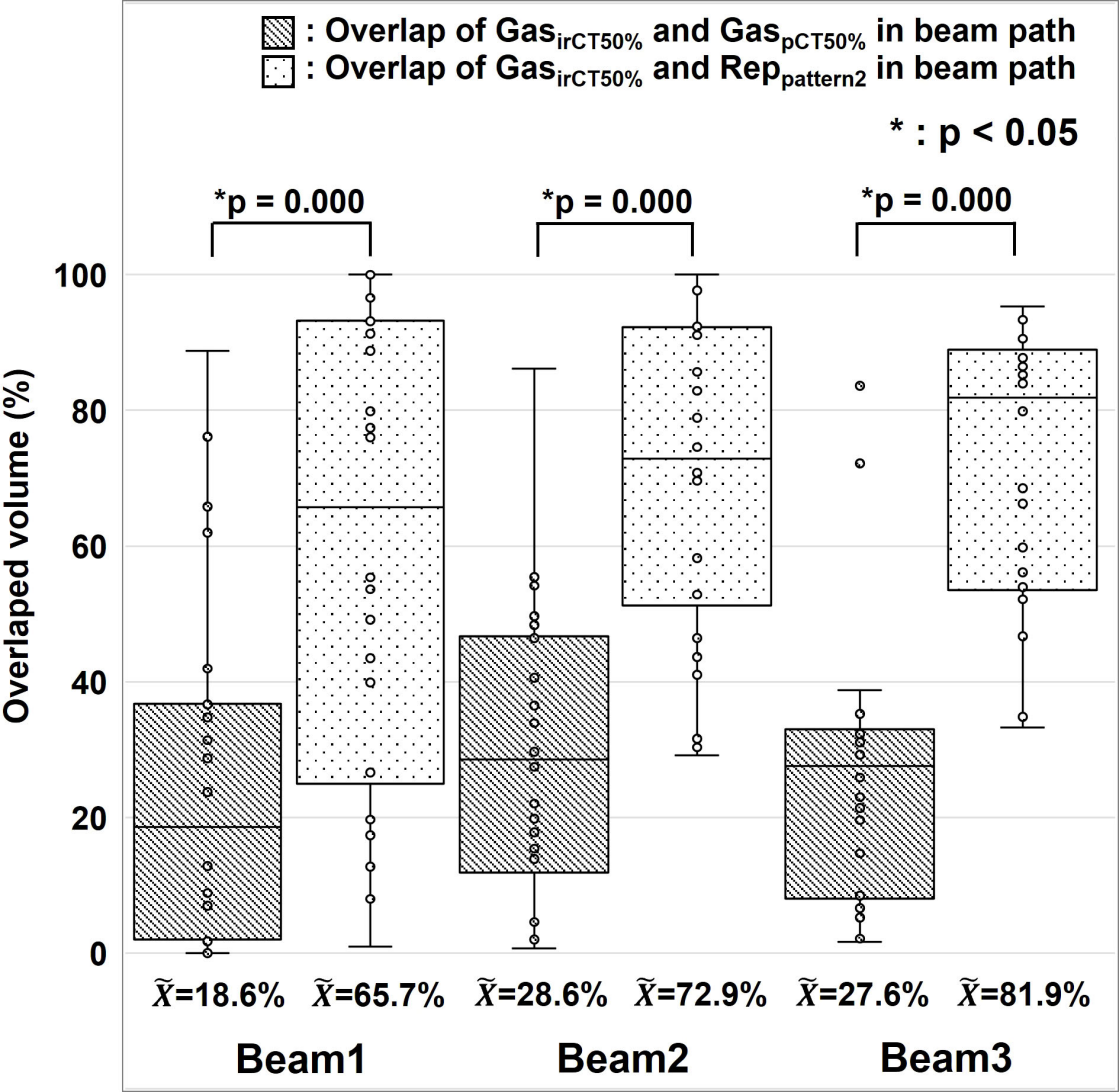
Positional changes of gastrointestinal gas. The median, first and third quartile, maximum and minimum values, and outliers for the concordance of gas contouring is presented in a box-and-whisker plot per each beam. The gas contour of the treatment planning CT images is presented as Gas_pCT50%_ , the gas contour in the in-room CT images is presented as Gas_irCT50%_ , and the replacement region set using Pattern 2 during treatment planning is presented as Rep_pattern2_. We used the Wilcoxon signed rank test for the statistical analysis. The symbol * indicate that there is a significant difference between them.

We analyzed the positional changes of G-gas and the validity of the replacement regions that correspond to those set with Pattern 2 (**Figure 2C**). On the premise that actual treatment will be performed using 2D–3D bone matching, pCT and irCT images were fused by bone matching using the MIM software, and we evaluated the concordance rate between the gas contour (Gas_pCT50%_ ) delineated on the pCT_50%_ images along the beam path and the gas contour (Gas_irCT50%_ ) delineated on the irCT_50%_ images as per volume. Furthermore, we evaluated the concordance rate between the replacement regions in Pattern 2 and Gas_irCT50%_ . **Figure 6** shows the resultant concordance rate. First, the median concordance rate between gas contouring along the path of each beam on Gas_pCT50%_ and Gas_irCT50%_ was 18.6%, 28.6%, and 27.6% in Beam 1, 2, and 3, respectively. Next, the median concordance rate between the replacement region of Pattern 2 and Gas_irCT50%_ was 65.7%, 72.9%, and 81.9% in Beam 1, 2, and 3, respectively. In the radiotherapy of the abdominal region, the position of G-gas was rarely consistent; however, the majority of gas remains mobile within the region that is considered as the gastrointestinal tract. The area replacement method of Pattern 2 is expected to minimize the effects of positional changes in G-gas throughout the treatment period and during irradiation (10).

The evaluation of variations between G-gas volume and *Δ*TC_CTV_ and *Δ*HI (**Figure 5**), showed that Pattern 2 was the least affected but Pattern 1 was the most affected by G-gas. In the initial treatment planning of Pattern 1, the beam stop positions were determined under the no-gas state. The effect of G-gas was considered the most significant due to the large rSPR value difference between treatment planning and each treatment. As shown in **Figure 6**, the concordance rate of G-gas position was lower in Pattern 3 than Pattern 2. However, Pattern 3 was able to consider the effect of G-gas more than Pattern 1, and the effect of G-gas was considered to be reduced compared with that in Pattern 1. Pattern 2 was able to consider variations in the location of G-gas more than Pattern 3 due to area replacement, and robustly responded to variations in the amount of G-gas due to mean value replacement.

**Figure 7:**
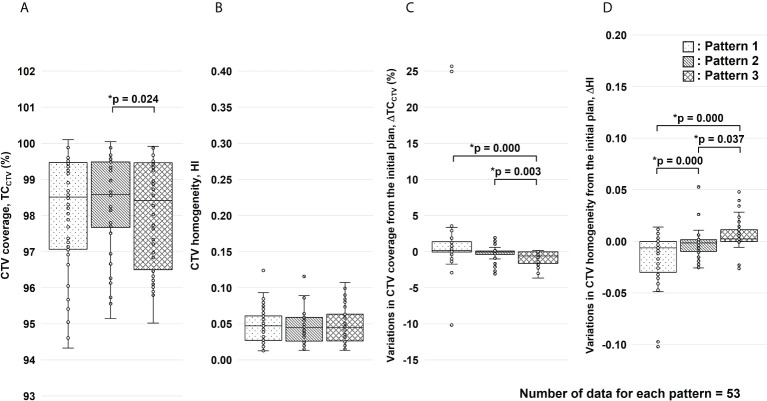
Results of the clinical target volume (CTV) coverage (TC_CTV_) and homogeneity index (HI) evaluation for the total dose. Figure panels **(A, B)** show the results of TC_CTV_ and HI, respectively. Figure panels **(C, D)** show the results of variations in TC_CTV_ (ΔTC_CTV_) and HI (ΔHI) from the initial plan, respectively. These results were corrected for changes in factors other than gastrointestinal gas. For each replacement pattern, the median, first and third quartiles, maximum and minimum values, and outliers for **(A)** TC_CTV_, **(B)** HI, **(C)** ΔTC_CTV_, and **(D)** ΔHI are presented in a box-and-whisker plot. Statistical analyses were performed using the Friedman test. The symbol * indicate that there is a significant difference between them.

Finally, we calculated a dose distribution of 55.2 Gy RBE/12 fractions (3, 30), which was adjusted for the effects of G-gas (**Figure 7**). Based on the results of this study, we propose that a treatment plan for robustness against G-gas can be prepared in which the fractions of Beams 1, 2, and 5 in the total dose are four, two, and six. The gantry angle and ratio of the irradiation dose were decided based on the effects of G-gas, uncertainty of the RBE model, and uncertainty of beam range calculation. Concerning Pattern 2, the variation was clearly significantly low for both ΔTC_CTV_ and ΔHI; compared with the other methods, the Pattern 2 mean value replacement method is feasible for robust treatment planning. The maximum dose, which covered 2 cm^3^ (D_2cm3_) of the gastrointestinal tract was 1.3–45.8 Gy RBE/12 fractions, and the dose constraints specified by the Japan Carbon-ion Radiation Oncology Study Group (3, 30) were satisfied. This study had some limitations. First, the number of patients evaluated was 10, and the number of irCT scans was 4–7 per patient during the treatment. Although irCT scans were not performed at every treatment, the total number of beams used for evaluation in this study was considered sufficient. However, the accuracy of the analysis may be improved by increasing the number of beams. Second, individual differences in G-gas volume may have occurred due to dietary restrictions, drinking water restrictions, and medications. Reducing the effects of G-gas may be possible by taking appropriate measures for each patient, but G-gas cannot be completely removed.

## Conclusions

This study demonstrated that treatment plans that were robust to changes in G-gas could be prepared by setting the replacement range as the region based on gastrointestinal contours delineated on pCT images and then replacing the range with the mean rSPR value obtained for each region. Our method improved dose delivery to the tumor. We are currently formulating treatment plans at our hospital based on this method. Despite the need for clinical follow-up, we believe that this method may help improve clinical outcomes. Furthermore, although this study focused on pancreatic cancer, this method might be used for particle beam scanning radiation for cancers that are affected by G-gas, such as cancers of the liver and abdominal cartilage, as well as gynecologic cancers. This method does not require any particular software or equipment, and it is simple to implement in clinical practice.

## Data availability statement

The original contributions presented in the study are included in the article/**Supplementary Material**. Further inquiries can be directed to the corresponding author.

## Ethics statement

The studies involving human participants were reviewed and approved by Institutional Review Board of Kanagawa Cancer Center (2019eki-106, August 30, 2021). The patients/participants provided their written informed consent to participate in this study.

## Author contributions

Conceptualization, YK and HK; Methodology, YK; Validation, YK, YM, YTakay, KI, TKu, and SMiy; Formal analysis, YK; Investigation, YK and HF; Resources, YK; Data curation, YK and HF; Writing–original draft preparation, YK; Writing–review & editing, HK, SMin, YM, YTakay, KI, TKu, SMiy, TKam, IS, YTakak, NM, KT, and DY; Visualization, YK; Supervision, YK and HK; Project administration, YK, HK, SMin, and DY; Funding acquisition, HK, SMin, and DY. All authors contributed to the article and approved the submitted version.

## Funding

This research was funded by Toshiba Energy Systems and Solutions Corporation (2019-Epi-102, 20 November 2019) and Japan Society for the Promotion of Science (JP20K08151).

## Acknowledgments

This work was supported by JSPS KAKENHI Grant Number JP20K08151. The authors would like to thank Anna D, Ph.D., from Enago (www.enago.jp) for the English language review.

## Conflict of interest

HF is employed by Accelerator Engineering Corporation. HK, SMin, and DY received research funding from Toshiba Energy Systems and Solutions Corporation.

The remaining authors declare that the research was conducted in the absence of any commercial or financial relationships that could be construed as a potential conflict of interest.

## Publisher’s note

All claims expressed in this article are solely those of the authors and do not necessarily represent those of their affiliated organizations, or those of the publisher, the editors and the reviewers. Any product that may be evaluated in this article, or claim that may be made by its manufacturer, is not guaranteed or endorsed by the publisher.
