# Supplementary material for: Robust treatment planning in scanned carbon-ion radiotherapy for pancreatic cancer: Clinical verification using in-room computed tomography images
Source: Front Oncol. 2022 Aug 29;12:974728. doi: 10.3389/fonc.2022.974728 (PMC9465304; doi:10.3389/fonc.2022.974728)
Supplement: Supplementary file 1 [file DataSheet_1.pdf]

Supplementary Material

Preliminary analysis (Methods and Results)

Changes in the patient's physique, gastrointestinal gas (G-gas), and tumor position were all considered as causes of decreased clinical target volume (CTV) coverage (TC_{CTV}). In ten cases of pancreatic cancer (Table 1), we preliminarily analyzed the factors that led to decreased TC_{CTV} during the treatment. The dose distributions for a total dose of 55.2 Gy relative biological effectiveness-weighted absorbed dose (RBE)/12 fractions [3,30] were calculated. The fractions of the gantry angles 0°, 165°, and 270° in the supine position to the total dose were four, six, and two, respectively. First, TC_{CTV} variation, including all factors (ΔTC_{CTV}), were calculated as follows:

$$\Delta TC_{CTV} = TC_{CTV,\, irCT} - TC_{CTV,\, Initial} \quad (1),$$

where $TC_{CTV,\, irCT}$ indicates $TC_{CTV,}$ calculated using in-room computed tomography (irCT) images taken on each treatment day and $TC_{CTV,\, Initial}$ indicates $TC_{CTV,}$ calculated using initial planning CT images. To evaluate the effects of changes in the patient's physique (ΔTC_{Sur}), irCT images adjusted to the patient's physique of the initial planning were prepared. The body size difference between the initial planning CT and irCT images was prepared as a contour. Compared to the initial planning CT image, thicker areas were replaced with the value of relative stopping power ratio (rSPR) for air and thinner areas with rSPR for fat. The rSPR is calculated using the CT value is equivalent to the CT value's replacement [24–29]. The dose distributions were calculated using the patient's physique adjusted. The TC_{CTV} differences before and after adjustment were calculated as follows:

$$\Delta TC_{Sur} = TC_{CTV,\, irCT} - TC_{CTV,\, irCT(Sur)} \quad (2),$$

where $TC_{CTV,\, irCT(Sur)}$ indicates TC_{CTV} on irCT images that were adjusted to the patient's physique at the initial planning. To evaluate G-gas (ΔTC_{Gas}) effects, the G-gas contours were drawn on the irCT images; contours were replaced with the rSPR values without G-gas. The dose distributions were calculated using replacement, and the difference in TC_{CTV} before and after replacement was calculated as follows:

$$\Delta TC_{Gas} = TC_{CTV,\, irCT} - TC_{CTV,\, irCT(Gas)} \quad (3),$$

where $TC_{CTV,\, irCT(Gas)}$ indicates TC_{CTV} on irCT images whose G-gas contours were replaced. Finally, the effect of changes in the tumor position (ΔTC_{Pos}) was calculated as follows:

$$\Delta TC_{Pos} = \Delta TC_{CTV} - \Delta TC_{Sur} - \Delta TC_{Gas} \quad (4)$$

In this section, we assumed ΔTC_{Sur}, ΔTC_{Gas}, and ΔTC_{Pos} as factors. However, ΔTC_{Pos} may include other factors that we do not expect.

The main factors were found to be changes in the tumor position and G-gas (Supplementary Figure 1). The effect of changes in the tumor position will be improved by target matching [12]. TC_{CTV} during the treatment period can be further improved if a robust treatment plan for G-gas can be prepared.

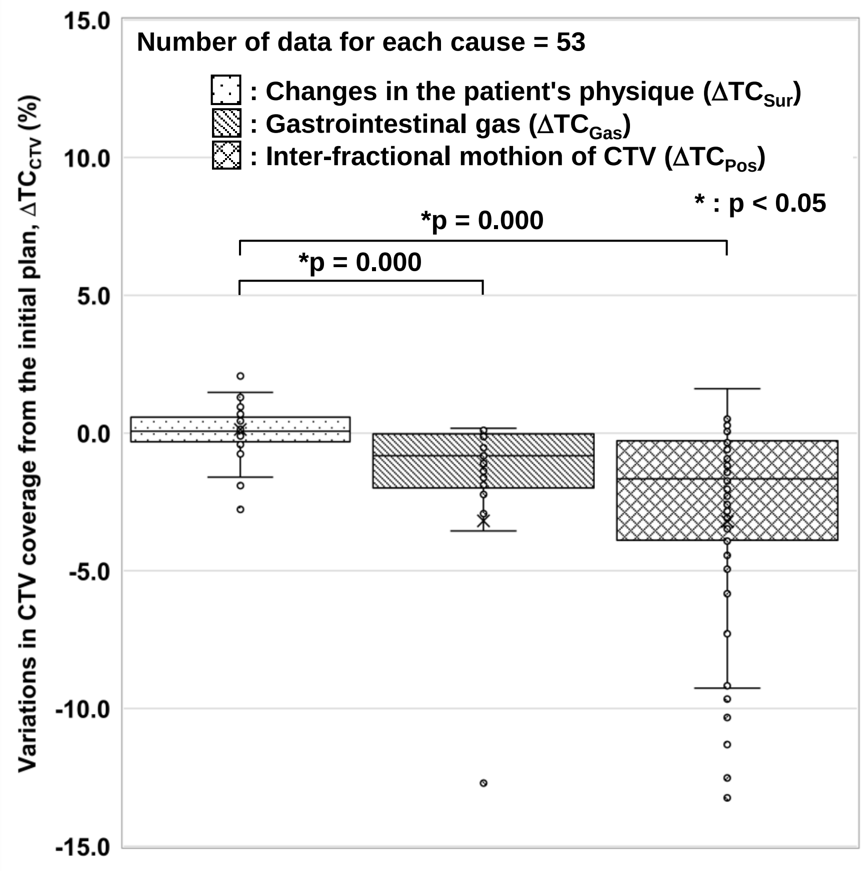

Supplementary Figure 1. Causes of decreased of clinical target volume (CTV) coverage (TC_{CTV}) in the pancreatic cancer treatment. The median, first, and third quartiles; maximum and minimum values; and outliers for the causes of TC_{CTV} decreases are presented in a box-and-whisker plot. The vertical axis presents the variations of TC_{CTV} (ΔTC_{CTV}) from the initial plan. Statistical analyses were then performed using the Friedman test.
